# Prof. Daniel Meeker

**Published:** 1856-08

**Authors:** 


### EDITORIAL DEPARTMENT.

### QUACKERY IN THE PROFESSION.

Our readers well know that we have repeatedly attempted an exposure of the various systems of quackery with which community is cursed, and can bear testimony, too, to the severity with which we have assailed them. Of the results, it is not for us to speak, but one assurance we can make, and that is that in this warfare in which we have engaged, with a virulence on our part which some may have thought unjustifiable, and quite too bitter, we have been actuated by a sincere desire to promote the best interests and well-being of society, and in defence of legitimate medicine. In exposing medical infidelity, charlatanism, and dishonesty, we did so, directed by moral considerations, assuming the ground that error should be exposed and wrong prevented, and in this work our conscience prompts to a continuance.

But it has been said by one whose authority no one will doubt, "that charity begins at home;" "that we should take the beam out of our own eyes that we may see the mote in others." This is a difficult task always, but in this case it is unpleasant and painful to expose the low means and measures of those who claim to be our brethren, whose position renders them only the more competent to bring reproach upon the regular profession, which is made to cover over and indirectly endorse their unblushing quackery.

The evil of which we complain is a growing one, and if not arrested by exposure and denounced when exposed, a longer tolerance will give it boldness, and familiarity will make it reconcilable. We say it is growing, and we mean by this, that it is not confined, as heretofore, to those who are in the ranks and recognised by the profession, and yet hold no prominent position therein, but it has reached to and is practiced by those who claim prominence and position, yea even to teachers and professors. Humiliating as the reflection is, it is manifestly too true, but fortunately the number is limited. To witness the bold and immodest irregularities, such as fall under our observation occasionally, nay frequently, is sufficient to awaken disgust in the mind, and is well calculated to mislead and betray those of less pretensions into similar errors, but in whom it might be overlooked, if such a thing was at all pardonable.

Merit and popular fame are now quite different things. Modest merit deigns to become truly popular, because of its capacity for usefulness. It relies upon application, industry, and unwearied effort for public favor and popular sentiment in its behalf. True merit and true greatness rest upon honest, persevering, and unremitting labor for the common weal, and in these efforts it is quiet, unobtrusive, and without display. Public sentiment arises from an observation of the good that follows its footsteps, which arises and diffuses itself abroad as the fragrance of the tiny and delicate flower concealed by the tall grass in which it flourishes. It is natural and pardonable in one who, after faithful service and long continued effort, has succeeded in his aims in behalf of his fellow man, and who secures thereby public esteem therefor, to feel grateful for public testimony. Not only is it pardonable in him, but it is but just on the part of community that it testifies in his favor. Such ambition on the one hand is just, and the open testimony on the part of the public is due in each instance, and serves as an incentive to others to follow the example.

But there is another class, who, regardless of these requisites, and contemning a process requiring both patience and labor, in order to secure public attention, seek every means, fair or dishonorable, indifferently careless, so that the goal of popular praise is secured. With them there is no difference between real merit and blind public accord, which has been won, no matter how, by chicanery, deceit, or management, so that the tide of public opinion is set fortunately in their direction.

They study well the susceptibility of the masses to being led by whims and caprices, and their talents for intrigue are brought into requisition in order to foist themselves into an ephemeral prominence. But no sooner has one trick or one course of policy lost its freshness, and their fame, thus dishonorably acquired, but now waning, than some new light is produced, or some new blaze is made to burn with such refulgence as to blind the credulous anew. If this cannot be accomplished, some new locality is selected and the old tricks revived and reproduced. It requires great resources of intrigue to sustain such for a long time in one locality, as the public mind takes "a sober second thought" occasionally, and when the mental vision is clear, the hollow artifice is detected. It must, therefore, be led on from one thing to another, made to follow this ignis fatuus, and that so that no time for calm discernment is allowed.

Their names must be kept before the public gaze, in accordance with their favorite maxim that to become conspicuous, they must become notorious. Their vanity prompts them to search out and discover all occasions for its gratification, and their shameless arrogance and unblushing boldness, lend their helps in securing them for their gratification. They fulminate their own praises or bribe others to its performance—their wondrous skill—their great advantages—their unparalleled success—their long residence in some great hospital—their tour abroad—their familiarity with distinguished savans—their several diplomas framed and hung up conspicuously—and the respectful attentions they have received from certain great characters on some conspicuous occasion—they are foreigners and pupils of the great masters of science abroad, and swell and puff their own consequence because of this fact, with an air of arrogance, as if the profession of this country were composed of ignoramuses. And then others parade their great surgical skill, by reports of operations never performed,—or if performed, have been done in a majority of instances, with open doors and a gaping throng drummed up to witness the trancendental performance. There is in our memory at this time, one of such sickening and disgusting spectacles, in which the operation was performed in a house upon a public street and an admiring crowd collected, and after looking upon the scene of blood, were to scatter off, bearing upon their lips each, heralds of his great surgical fame. Retributive justice followed the operator in hot pursuit, for the operation was not *absolutely* necessary and his patient died soon afterward.

Would these nauseating spectacles have been seen or tolerated in the days of Rush, Physick, Porrish, Potter, and others of the bright luminaries of the profession in the early period of the history of this country? No, they were content in those days with the hope that their labors and services in behalf of suffering humanity should speak for themselves, which course of conduct gave dignity to the profession and honor to the calling.

As journalists, we have felt it our duty to call the attention of the profession to a closer observance of our code of ethics, and call upon each to expose to the reprehension of the profession, every act of low sycophancy or charlatanry on the part of those who claim a rank among us. There are other points of objection which we would notice now, but this article has exceeded the bounds contemplated, and we must resume the subject in the future.

### MEDICAL WORKS.

Owing to the great removal of the profession in this State, from the eastern cities, whence emanates our medical literature, much inconvenience has been experienced in the prompt procurement of new and recent works. To remedy this difficulty, we have been in correspondence with some of the principal publishing houses in Philadelphia, but as no arrangement has been effected, we would say to our medical friends in the interior who have conferred with us on the subject, that Mr. Robert Ogden, Bookseller, of this place, has recently received a large supply of standard medical works, and will be receiving further additions from time to time. Arrangements can be effected with him also for the procurement of any new work not in his possession. We have looked over his list and find the catalogue not only large, but the works approved standard authorities. We can cheerfully recommend him to the profession as an accommodating, honorable gentleman, and in every way disposed to aid medical men in their efforts to procure fresh medical works. We would say, then, to those who are in want, to address him on the subject, and we are assured they will receive prompt attention.

### PROF. DANIEL MEEKER, M. D.

The profession of this region will doubtless be much gratified in being informed that the gentleman whose name stands at the head of this article, has accepted the appointment of Professor of Anatomy in this institution.

Prof. Meeker is an old and experienced teacher, and occupies a most exalted position in his profession in the State of Indiana. He is devoted to the cultivation of medical science, which he has cultivated with an assiduity and industry which has allowed no relaxation, and which, too, has placed his name most prominently before the medical public.

He is one of the best teachers of the branch assigned him in this institution, in the West, and is an interesting, engaging and impressive lecturer. He will come with a determination and a will to give a most thorough course upon that branch the ensuing winter. The Faculty are much gratified in being able to say to those who will attend with us the coming session, that one so distinguished as a teacher, and one who ranks so deservedly high as a physician and a gentleman, will occupy during the term, so important a chair as that of Anatomy.

